# An *Arabidopsis* mitochondria-localized RRL protein mediates abscisic acid signal transduction through mitochondrial retrograde regulation involving ABI4

**DOI:** 10.1093/jxb/erv356

**Published:** 2015-07-10

**Authors:** Xuan Yao, Juanjuan Li, Jianping Liu, Kede Liu

**Affiliations:** National Key Laboratory of Crop Genetic Improvement, Huazhong Agricultural University, Wuhan 430070, China

**Keywords:** ABA signalling, ABI4, AOX1a, mitochondria, mitochondrial retrograde regulation RRL.

## Abstract

The molecular mechanisms of abscisic acid (ABA) signalling have been studied for many years; however, how mitochondria-localized proteins play roles in ABA signalling remains unclear. Here an *Arabidopsis* mitochondria-localized protein RRL (RETARDED ROOT GROWTH-LIKE) was shown to function in ABA signalling. A previous study had revealed that the *Arabidopsis* mitochondria-localized protein RRG (RETARDED ROOT GROWTH) is required for cell division in the root meristem. RRL shares 54% and 57% identity at the nucleotide and amino acid sequences, respectively, with RRG; nevertheless, RRL shows a different function in *Arabidopsis*. In this study, disruption of *RRL* decreased ABA sensitivity whereas overexpression of *RRL* increased ABA sensitivity during seed germination and seedling growth. High expression levels of RRL were found in germinating seeds and developing seedlings, as revealed by β-glucuronidase (GUS) staining of ProRRL–GUS transgenic lines. The analyses of the structure and function of mitochondria in the knockout *rrl* mutant showed that the disruption of *RRL* causes extensively internally vacuolated mitochondria and reduced ABA-stimulated reactive oxygen species (ROS) production. Previous studies have revealed that the expression of alternative oxidase (AOX) in the alternative respiratory pathway is increased by mitochondrial retrograde regulation to regain ROS levels when the mitochondrial electron transport chain is impaired. The APETALA2 (AP2)-type transcription factor ABI4 is a regulator of *ALTERNATIVE OXIDASE1a* (*AOX1a*) in mitochondrial retrograde signalling. This study showed that ABA-induced *AOX1a* and *ABI4* expression was inhibited in the *rrl* mutant, suggesting that RRL is probably involved in ABI4-mediated mitochondrial retrograde signalling. Furthermore, the results revealed that ABI4 is a downstream regulatory factor in RRL-mediated ABA signalling in seed germination and seedling growth.

## Highlight

This study reports that an *Arabidopsis* mitochondria-localized RRL protein positively regulates ABA signalling through downstream regulatory transcription factor ABI4-mediated mitochondrial retrograde regulation during seed germination and seedling growth.

## Introduction

The phytohormone abscisic acid (ABA) regulates many important aspects of plant development, such as promotion of seed maturation and dormancy and inhibition of the transitions from the embryonic to the germinative phase (Rock, 2000; Finkelstein *et al.*, 2002; Hirayama and Shinozaki, 2007). ABA is also a key endogenous signal to help plants adapt to various biotic and abiotic stresses (Rock, 2000; Shinozaki and Yamaguchi-Shinozaki, 2000). Understanding of ABA signalling has progressed greatly, and a recent breakthrough is the discovery of the ABA perception mechanism in the primary ABA signal transduction pathway (Raghavendra *et al.*, 2010). ABA binds to the receptor PYR1/PYLs/RCARs, which together inactivate type 2C protein phosphatases (PP2Cs) such as ABI1 and ABI2. Inactivation of the protein phosphatases enables the release of active OST1 and related SNF1-type kinases (SnRKs) via PP2C inactivation to initiate ABA signal transduction (Fujii *et al.*, 2009; Miyazono *et al.*, 2009; Nishimura *et al.*, 2009). OST1 actives the anion channel SLAC1 (Geiger *et al.*, 2009; Lee *et al.*, 2009) and inhibits the cation channel KAT1 (Sato *et al.*, 2009) by phosphorylation to induce stomatal closure. In ABA signalling to the nucleus, OST1 and related SnRKs target ABA-responsive element-binding factor (ABFs/AREBs) such as the basic leucine zipper (bZIP) transcriptional factor ABI5 (Finkelstein *et al.*, 2005) to regulate ABA-responsive gene expression. ABI3 is a B3 transcriptional factor which binds to ABI5 to facilitate ABI5 binding to ABA-responsive elements (ABREs) (Nakamura *et al.*, 2001). In addition, ABI4, an APETALA2 (AP2)-type transcription factors binds the CE1 *cis*-element in the promoter of target genes in ABA signalling (Woodson and Chory, 2008).

ABI4 plays essential positive roles in the ABA biogenesis and signal transduction pathway, especially during seed dormancy and germination (Finkelstein *et al.*, 1998; Soderman *et al.* 2000; Shu *et al.*, 2013). *abi4* was identified as an ABA-insensitive mutant during seed germination (Finkelstein *et al.*, 1998), whereas overexpression of *ABI4* represses seed germination when exogenous ABA is applied (Holdsworth *et al.*, 2008). The mRNA level of *ABI4* is high in developing seed and relatively low in the vegetative tissues, and ABI4 protein is degraded through the 26S proteasomal pathway from the germination and seedling establishment stages (Finkelstein *et al.*, 2011). Moreover, an increasing number of findings have demonstrated that ABI4 is a versatile regulatory factor. ABI4 is found to regulate diverse processes including lipid biosynthesis and breakdown (Penfield *et al.*, 2006), redox regulation (Pontier *et al.*, 2007), cell wall metabolism (Talboys *et al.*, 2011), lateral root development (Shkolnik-Inbar and Bar-Zvi, 2010), salt responses (Quesada et al, 2000), nitrogen deficiency response (Yang *et al.*, 2011), sugar signalling (Arenas-Huertero *et al.*, 2000; Laby *et al.*, 2000), and mitochondrial and chloroplast retrograde signalling (Koussevitzky *et al.*, 2007; Giraud *et al.*, 2009; Sun *et al.*, 2011). High sugar levels lead to delayed seed germination (Dekkers *et al.*, 2004), slow seedling growth, impaired chloroplast development, and pale cotyledons. However, *abi4* mutants are insensitive to 6% glucose, lacking all the characteristics of sugar-directed arrested growth (Arenas-Huertero *et al.*, 2000; Huijser *et al.*, 2000). Elevated ABI4 mRNA levels result in glucose oversensitivity (Teng *et al.*, 2008; Cui *et al.*, 2012), which suggests that glucose promotes the sugar-directed growth arrest by the accumulation of ABI4 protein in young seedlings. Furthermore, ABI4 also functions as a point of convergence for anterograde (nuclear to mitochondrial) and retrograde (mitochondrial to nuclear) signalling pathways (Giraud *et al.*, 2009; Ng *et al.*, 2014). ABI4 mediates mitochondrial retrograde signals through regulation of the expression of *ALTERNATIVE OXIDASE1a* (*AOX1a*), which is a marker gene of mitochondrial retrograde regulation. ABI4 binds to the CE1 *cis*-element in the promoter of *AOX1a* and represses *AOX1a* gene expression. However, the repression due to the binding of ABI4 can be derepressed by ABA signal (Giraud *et al.*, 2009). The accumulation of *AOX1a* increases mitochondrial reactive oxygen species (mtROS) levels to keep the balance of the redox status and generate ROS signalling by mitochondrial retrograde regulation when the mitochondrial electron transport chain (mETC) is inhibited in plant cells (Popov, 2003; Ng *et al.*, 2014).

Mitochondria are responsible for both ATP and mtROS production in cells, and the mechanisms of mitochondrial biogenesis and function are relatively well understood. However, there is little knowledge of the regulatory factors involved in the various developmental processes and signalling pathways in mitochondria, which calls for more research on the biological role of mitochondria-localized protein at a molecular level (Ng *et al.*, 2014). A previous study discovered that the *Arabidopsis RETARDED ROOT GROWTH* (*RRG*) gene functions in the regulation of cell division in the root meristem (Zhou *et al.*, 2011). RRG is a mitochondria-localized protein containing a conserved DUF155 domain of unknown function. Disruption of *RRG* causes retarded root growth because of reduced numbers of dividing cells, the rate of cell production, and endoreduplication in the root meristem. Here, the function of another *Arabidopsis* mitochondria-localized protein with a DUF155 domain named RRL (*RETARDED ROOT GROWTH-LIKE*) in ABA signalling was characterized. RRL plays a positive role in ABA signal transduction in seed germination and primary root growth with regulation of the expression of the AP2-type transcription factor ABI4 and ABI4-meditated mitochondrial retrograde signalling.

## Materials and methods

### Plant materials and growth conditions

*Arabidopsis thaliana* ecotype Columbia (Col-0) was used as the wild-type control in this study. *rrl* (SALK_022878) and *abi4-1* (CS8104) were from the Arabidopsis Biological Resource Center (ABRC; http://www.arabidopsis.org/abrc/). Seeds were surface sterilized and plated on MS medium (Murashige and Skoog, 1962) containing 0.8% (w/v) agar and 1% (w/v) sucrose. All plates were vernalized at 4 °C for 2 d in the dark followed by incubation under a 16h light/8h dark photoperiod and 70% humidity at 23 °C. For germination and seedling growth assay, ABA (Sigma, USA) was added to the medium.

### Plasmid constructions

For complementation assay, a 4.0kb genomic DNA fragment including upstream, coding, and downstream regions of the *RRL* gene was cloned into the binary vector pCAMBIA3301 at the *BamH*I/*Pst*I cloning sites to construct the ProRRL-RRL plasmid. In order to generate the RRL-OE plasmid and overexpress *RRL* in plants, the 1.2kb full-length cDNA of the *RRL* gene was inserted into the *Nco*I/*Pml*I cloning sites of pCAMBIA3301 to replace the β-glucuronidase (GUS) gene driven by the *Cauliflower mosaic virus* 35S promoter. To make the plasmid ProRRL–GUS for analyses of the expression pattern, a 2kb *RRL* promoter fragment was cloned into the binary vector pBI101–GUS at the *Kpn*I/*Pst*I cloning sites. To detect subcellular localization of RRL, the plasmid RRL–GFP was constructed by cloning the RRL full-length cDNA sequence in the *Sal*I/*Pst*I cloning sites of vector p35S-SUNGFP, inserting the *RRL* gene in the N-terminus of the green fluorescent protein (GFP). The sequences of primers used for plasmid construction are listed in Supplementary Table S1 available at *JXB* online.

### Generation of transgenic plants, double mutant *abi4-1 rrl*, and hybrid *abi4-1 OE-4*

*Agrobacterium tumefaciens* strain GV3101 harbouring the plasmid RRL–GFP was used to transform *Arabidopsis* wild-type (Col-0) plants by floral dip-mediated infiltration (Clough and Bent, 1998) in order to generate RRL–GFP transgenic plants for subcellular localization analysis. For the generation of *RRL*-overexpression transgenic plants, *Agrobacterium* GV3101 harbouring the plasmid RRL-OE was used to transform the Col-0 plants. The transgenic lines carrying plasmids ProRRL-RRL and ProRRL–GUS were also generated in the Col-0 background for complementation assay and expression pattern determination, respectively. Homozygous T_3_ transgenic lines were selected for analyses. Double mutant *abi4-1 rrl* and hybrid *abi4-1 OE-4* lines were generated by crossing the mutant *abi4-1* (maternal) with *rrl* (paternal) and the *RRL*-overexpression transgenic line *OE-4* (paternal), respectively. The *abi4-1* mutant contained a G-deletion mutation at base pair 619 that causes early termination of ABI4 translation (Finkelstein *et al.*, 1998). A dCAPS (derived cleaved amplified polymorphic sequence) marker (amplified by primers ABI4dcapsFP and ABI4dcapsRP) was designed to identify homozygous *abi4-1* plants from the F_2_ population derived from the cross between *abi4-1* and *rrl* mutants. PCR products from individual F_2_ plants were digested by *NIa*IV. The sequences of ABI4dcapsF and ABI4dcapsR are listed in Supplementary Table S2 at *JXB* online. Homozygous F_2_ plants were used for further study.

### RT-PCR analyses

Semi-quantitative real-time PCR (RT-PCR) was performed to analyse the expression of *RRL*. Total RNA was isolated from 5-day-old seedlings using an RNAprep Pure Plant kit (Bio TeKe, China) supplemented with the RNase-free DNase I set, according to the manufacturer’s instructions. RNA samples were reverse transcribed with a ReverAid First Strand cDNA Synthesis Kit (Thermo Scientific, USA). The expression level of the *ACTIN2* gene (AT3G18780) was used as a loading control. Quantitative RT-PCR analysis was performed to assay expression levels of mitochondria-associated and ABA-responsive genes with or without ABA treatment. The cDNA was amplified using a SYBR Green master mixture (Bio-Red, USA) with a LightCycler 96 (Roche, USA). Amplification of the *β-ACTIN8* (AT1G49240) gene was used as an internal control. The sequences of primers used for semi-quantitative and quantitative RT-PCR analyses are listed in Supplementary Table S2 at *JXB* online.

### Histochemical analysis and subcellular localization analysis

GUS staining was performed to determine the expression pattern of RRL in the ProRRL–GUS transgenic lines. Various tissues of transgenic seedlings were incubated with 5mM K_4_Fe(CN)_6_, K_3_Fe(CN)_6_, 0.2mM PBS buffer (KH_2_PO_4_ and K_2_HPO_4_, pH 7.0), 0.1% Triton X-100, 0.5mg ml^–1^ X- Gluc (Sigma, USA) at 37 °C overnight and washed with 70% ethanol three times (Jefferson *et al.*, 1987). A stereomicroscope (Nikon, smz1000, Japan) was used for examination and photography. To determine the subcellular localization of RRL, leaves of RRL–GFP transgenic *Arabidopsis* plants were stained with a mitochondrion-selective probe MitoTracker Red (fluorescent dye) (CMTMRos; Invitrogen) following the instruction manual. A transient expression experiment was also carried out to analyse the subcellular localization of RRL as described previously by Voinnet *et al.* (2003). *Agrobacterium* GV3101 cells were transformed with the plasmid RRL–GFP. The transformed cells were harvested and resuspended in 10mM MES-KOH, pH 5.6, containing 10mM MgCl_2_ and 150mM acetosyringone, and then mixed with *Agrobacterium* expressing the silencing suppressor p19 of *Tomato bushy stunt virus* (Voinnet *et al.*, 2003) to a final optical density at 600nm (OD_600_) of 0.8. After incubation for 3h at room temperature, the *Agrobacterium* suspension was injected into expanded leaves of 4-week-old tobacco plants (*Nicotiana benthamiana* cv. SR1). Leaves were observed after 3–4 d with a laser scanning confocal imaging system (TCS SP2, Leica).

### Transmission electron microscopy

The ultrastructure of mitochondria was observed by transmission electron microscopy. The cut roots from 5-day-old *Arabidopsis* seedlings were fixed and rinsed in phosphate buffer containing 5% glutaraldehyde and 0.1M cacodylate (pH 7.4), and then were post-fixed in 1% osmium tetroxide for 2.5h at 5 °C. The fixed samples were embedded in catalysed epon (TAAB resin; Energy Beam Sciences), and polymerized at 65 °C for 3 d after dehydration in a graded concentration of acetone. Ultrathin sections were cut with a Reichert Ultracut S ultramicrotome and collected on mesh copper. Uranyl acetate was used to stain the sections, and the stained ultrathin sections were observed with an H-7650 transmission electron microscope (Hitachi).

### Detection of ROS

ROS production was detected using the fluorescent dye dichlorofluorescein (DCF) (Pei *et al.*, 2000). In order to quantify ABA-induced ROS levels, 5-day-old seedlings were loaded with 50 μM 2′,7′-dichlorodihydrofluorescein diacetate (H_2_DCFDA) for 30min and washed with ddH_2_O before ABA treatment. The loaded seedlings were then treated with 50 μM ABA (Sigma) for 1h. All the pictures were taken by a laser scanning confocal imaging system (TCS SP2, Leica) with excitation at 488nm and emission at 525nm to detect DCF fluorescence. The fluorescent images were analysed with Image J software (National Institutes of Health, USA).

## Results

### RRL (RETARDED ROOT GROWTH-LIKE) is an *Arabidopsis* mitochondria-localized protein like RRG (RETARDED ROOT GROWTH)

**Fig. 1.**
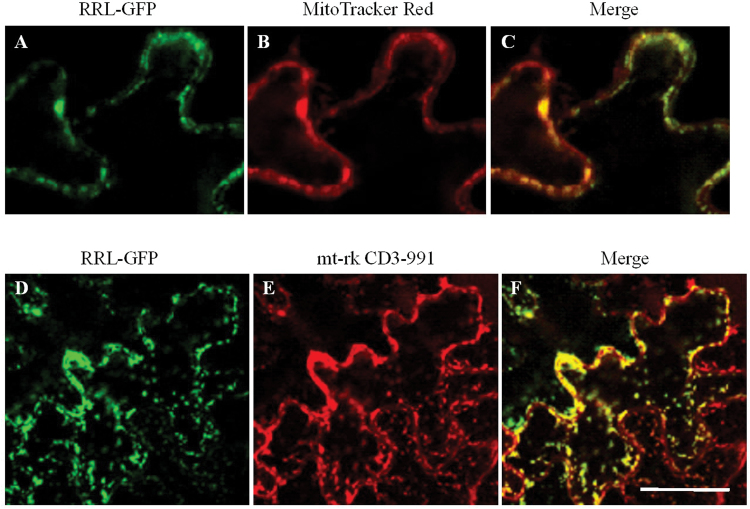
Mitochondrial localization of the RRL–GFP fusion protein. (A–C) Subcellular localization of RRL–GFP fusion protein in leaf epidermal cells of *Arabidopsis*. (A) RRL–GFP, (B) MitoTracker Red staining, (C) co-localization of RRL–GFP and MitoTracker Red. Scale bar=10 μm. (D–F) Subcellular localization of RRL–GFP fusion protein in leaf epidermal cells of tobacco. (D) RRL–GFP, (E) mt-rk CD3-991 (a mitochondrial marker). (F) The overlay image (merge) shows co-localization of RRL–GFP and the mitochondria marker. Scale bar=30 μm. (This figure is available in colour at *JXB* online.)

A previous study revealed that an *Arabidopsis* mitochondria-localized protein RRG (At1G69380) with a domain of unknown function (DUF155) is required for cell division in the root meristem. Disruption of *RRG* causes the reduction of dividing cells, the rate of cell production, and endoreduplication, which thus decreases the meristem size and root growth rate (Zhou *et al.*, 2011). At5G13610 is a homologous gene of At1G69380 and encodes another protein containing the DUF155 domain. At5G13610 shares 54% and 57% identity in nucleotide and amino acid sequences, respectively, with RRG (At1g69380) (see Supplementary Fig. S1A at *JXB* online) and was thus named *RETARDED ROOT GROWTH-LIKE* (*RRL*). The *RRL* gene is predicted to encode a novel protein with 402 amino acids (http://www.arabidopsis.org/servlets/TairObject?type=aa_sequence&id=1009134071). The RRL protein contains a conserved domain of unknown function (DUF155) and a putative C-terminal transmembrane domain (http://smart.embl-heidelberg.de/smart/show_motifs.pl) (see Supplementary Fig. S1B). To explore the subcellular localization of RRL protein, transgenic plants were generated with construct RRL–GFP in *Arabidopsis*. Fluorescence analyses of the expression of RRL–GFP revealed small punctate structures in leaf epidermal cells of the transgenic plants, which appeared to be localized to mitochondria. The mitochondrial localization of RRL–GFP was further confirmed by co-localization with a mitochondrion-selective dye MitoTracker Red (Fig. 1A–C). Additionally, the mitochondrial localization of RRL–GFP was also determined by tobacco leaf infiltration experiments with a well-known mitochondria marker, mt-rk CD3-991 (Nelson *et al.*, 2007), which was generated with a red fluorescent protein, mCherry (Shaner *et al.*, 2004). The RRL–GFP fusion protein co-localized with mt-rk CD3-991 by fluorescence analyses (Fig. 1D–F). These results showed that RRL is localized to mitochondria like RRG (Zhou *et al.*, 2011; Fig. 1).

**Fig. 2.**
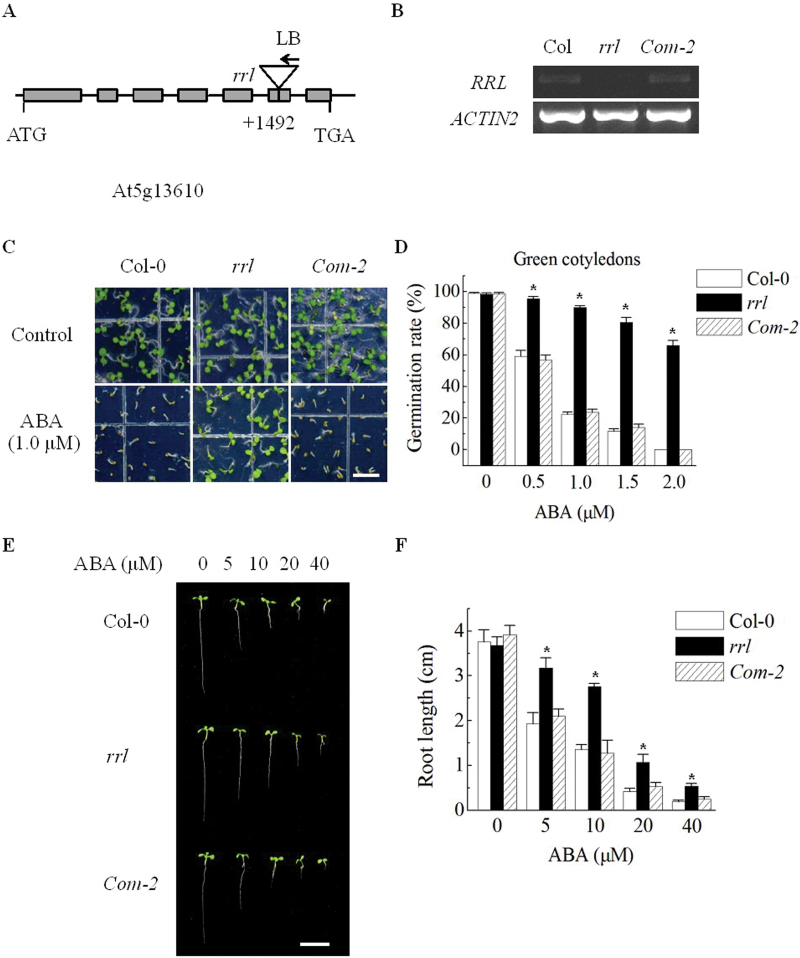
ABA-insensitive phenotype of the *rrl* knockout mutant in seed germination and seedling growth. (A) Schematic drawing (not to scale) of T-DNA insertion sites in the loss-of-function mutant allele of the *RRL* gene (At5G13610). Grey boxes and lines represent exons and introns, respectively. Arrowheads indicate orientations of T-DNA inserts. (LB, left border primer for T-DNA insertion; ATG, translation start codon; TGA, translation stop codon). (B) Semi-quantitative RT-PCR analysis of *RRL* gene expression in 5-day-old seedlings of Col-0 (wild type), the *rrl* mutant, and the *Com-2* line (complementation line). *ACTIN2* (AT3G18780) was used as the internal control. (C) Seeds were sown on MS medium supplemented or not with 1.0 μM ABA. Photographs were taken at day 5 after stratification. Scale bar=0.5cm. (D) Seeds were sown on MS medium supplemented with 0, 0.5, 1.0, 1.5, or 2.0 μM ABA. Germination rates (%) were analysed at day 5 after stratification. Values are the means ±SE of three independent experiments (*n*=100). *P<0.05 compared with Col-0 control. (E) Seedlings grew for 10 d after transfer from MS medium to MS medium supplemented with 0, 5, 10, 20, or 40 μM ABA. Seedlings were transferred from ABA-free medium to ABA-containing medium 48h after stratification. Scale bar=1cm. (F) Primary root lengths were assayed at day 10 after transfer. Values are the means ±SE of three independent experiments (*n*=50). *P<0.05 compared with Col-0 control. (This figure is available in colour at *JXB* online.)

To test if this gene has a similar function to *RRG*, a null mutant (SALK_022878C) with the T-DNA inserted in the sixth exon of the *RRL* gene (+1492) was obtained from the ABRC (Fig. 2A). At5G13610 transcripts were detected in the wild type (Col-0) but not in the *rrl* mutant (Fig. 2B), suggesting that *rrl* is a null mutant. The *rrl* mutants showed normal root growth like the wild type (Col-0) plants (see Supplementary Fig. S2 at *JXB* online), which is different from the retarded root growth of *rrg* mutants. Moreover, introduction of *RRL* cDNA driven by the *RRG* promoter could not recover the retarded root growth of the *rrg* mutant in complementation assays (see Supplementary Fig. S2). These findings indicated that RRL has different roles in mitochondria in *Arabidopsis*.

### Disruption of RRL decreases ABA responsiveness in seed germination and primary root growth

In order to predict the function of *RRL*, the sequence of the *RRL* promoter was analysed using PLACE (http://www.dna.affrc.go.jp/PLACE/signalup.html). Several ABA-responsive element- (ABRE-) related motifs were found in the promoter region of *RRL*. Therefore, the relationship between RRL and the ABA signalling pathway was investigated. To test the response of the *rrl* mutant to ABA, Col-0 and *rrl* seeds were sown on MS medium supplemented with 1% sucrose and 1.0 μM ABA. More than 90% of *rrl* seeds are able to germinate, and grow green and open cotyledons, whereas the germination of Col-0 seeds was inhibited at day 5 after stratification (Fig. 2C). Next the germination rates of the *rrl* mutant on MS medium containing 1% sucrose and a range of ABA concentrations (0, 0.5, 1.0, 1.5, and 2.0 μM ABA) was assayed by counting seedlings with green cotyledons (Fig. 1D). As shown in Fig. 2C and D, the *rrl* mutant was more insensitive to ABA than Col-0 in terms of seed germination. For example, ~66% (63–69%) of *rrl* seeds germinated and grew green cotyledons; in contrast, none of the Col-0 seeds germinated at day 5 after stratification when sown on MS medium supplemented with 2.0 μM ABA. In addition, ABA also inhibits primary root elongation during seedling growth as well as seed germination (Finkelstein *et al.*, 2002). Therefore, a primary root growth assay was used to investigate the ABA response of seedling growth. To assess ABA’s effect on seedling growth of the *rrl* mutant, germinating seed was transferred 48h after stratification from the ABA-free MS medium to MS medium containing 0, 5, 10, 20, or 40 μM ABA for 10 d. In these assays, stronger ABA inhibition of primary root elongation was observed in Col-0 seedlings than in ther *rrl* mutant when the ABA concentration was >5 μM (Fig. 2E, F).

In order to determine whether the ABA-insensitive phenotypes of the *rrl* mutant were caused by disruption of the *RRL* gene, the full-length cDNA was transformed under the control of its native promoter into *rrl* mutant plants. Thirteen independent transgenic lines were obtained. RT-PCR analysis indicated that the *RRL* gene was expressed after introducing the *RRL* gene into the *rrl* mutant (Fig. 2B) and the expression of *RRL* could complement the ABA-insensitive phenotype of the *rrl* mutant in both seed germination (Fig. 2C, D) and root growth assays (Fig. 2E, F). Taken together, these results suggest that disruption of *RRL* leads to the reduced response to ABA of seed germination and primary root growth.

### Overexpression of RRL increases ABA sensitivity of seed germination and primary root growth

**Fig. 3.**
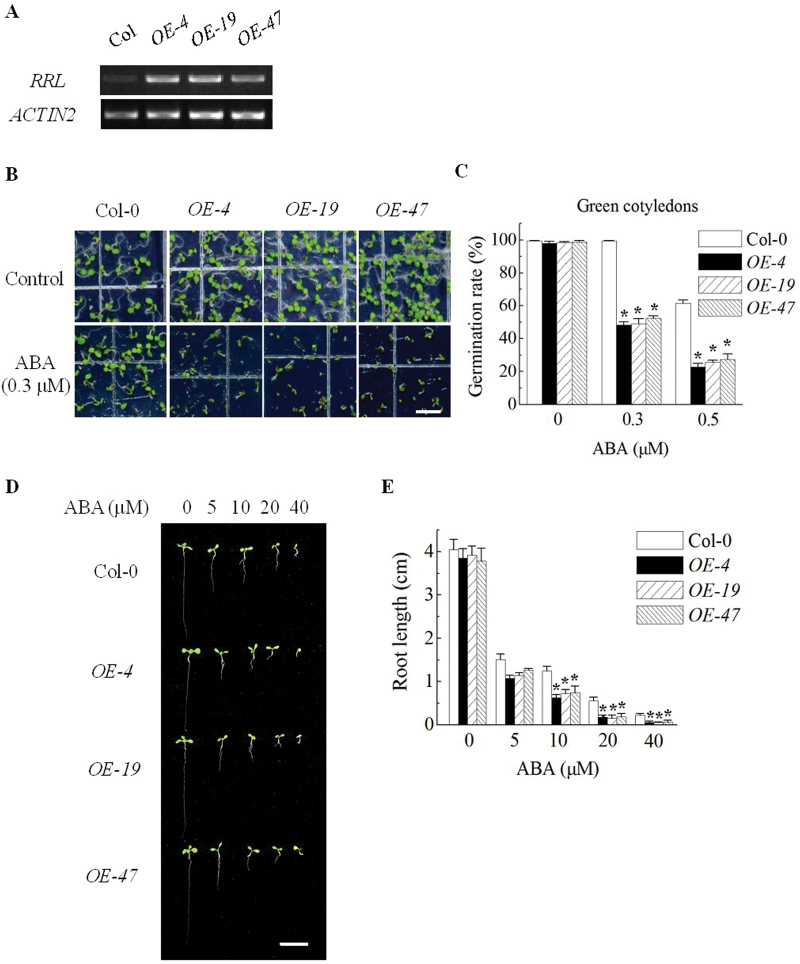
Overexpression of *RRL* cause the ABA-hypersensitive phenotype in seed germination and seedling growth. (A) Semi-quantitative RT-PCR was performed to assay expression levels of the *RRL* gene in 5-day-old seedlings of Col-0, *OE-4*, *OE-19*, and *OE-47* overexpression lines. *ACTIN2* gene expression represents the loading control. (B) Seeds were sown on MS medium supplemented or not with 0.3 μM ABA. Photographs were taken at day 5 after stratification. Scale bar=0.5cm. (C) Seeds were sown on MS medium supplemented with 0, 0.3, or 0.5 μM ABA. Germination rates (%) were analysed at day 5 after stratification. Values are the means ±SE of three independent experiments (*n*=100). *P<0.05 compared with Col-0 control. (D) Seedlings grew 10 d after transfer from MS medium to MS medium supplemented with 0, 5, 10, 20, or 40 μM ABA. Seedlings were transferred from ABA-free medium to ABA-containing medium 48h after stratification. Scale bar=1cm. (E) Primary root lengths were assayed at day 10 after transfer. Values are the means ±SE of three independent experiments (*n*=50). *P<0.05 compared with Col-0 control. (This figure is available in colour at *JXB* online.)

Fifty independent transgenic plants overexpressing *RRL* in the Col-0 background were generated. Homozygous T_3_ lines (*OE-4*, *OE-19*, and *OE-47*) with higher levels of *RRL* transcripts were selected and their responses to ABA were investigated (Fig. 3A). Seed germination and primary root growth assays were carried out in these overexpression lines to examine their responses to ABA. Taking *OE-4* as an example, only ~48% (46–50%) and ~23% (20–25%) of *OE-4* seeds germinated and grew green cotyledons, whereas the germination rate of Col-0 seeds was ~99% (99–100%) and ~61% (59–64%) when germinated on MS medium supplemented with 1% sucrose and 0.3 μM or 0.5 μM ABA at day 5 after stratification, respectively. (Fig. 3B, C). In root growth assays, the growth of primary roots was significantly inhibited in MS medium containing >10mM ABA compared with that of Col-0 seedlings. These results suggest that overexpression of *RRL* caused the increased response to ABA of seed germination and primary root growth.

Because the *rrl* mutant and the *RRL*-overexpression lines showed decreased and increased ABA sensitivity, respectively (Figs 2, 3), it was next determined whether disruption or overexpression of *RRL* would alter the plant response to drought stress. Two-week-old plants were deprived of water for 14 d and then rewatered. The wilting plants of Col-0, the mutant *rrl*, or the overexpression line *OE-4* could not recover by rehydration (see Supplementary Fig. S3A at *JXB* online). The transpirational water loss of detached rosette leaves was also measured on the 4-week-old Col-0, the mutant *rrl*, and the overexpression line *OE-4*. No significantly different water loss was found during 0–6h (see Supplementary Fig. S3B). A stomatal bioassay was also perfomed and it was found that there was no significant difference in ABA-induced stomatal closure of the three lines (see Supplementary Fig. S3C). Moreover, the *rrl* mutant displayed normal development of guard cells compared with Col-0 (see Supplementary Fig. S4). These results showed that Col-0, *rrl*, and *OE-4* displayed a similar response to drought stress, suggesting that *RRL* is not involved in ABA-dependent drought signal transduction.

### High expression of RRL in germinating seeds and developing seedlings

**Fig. 4.**
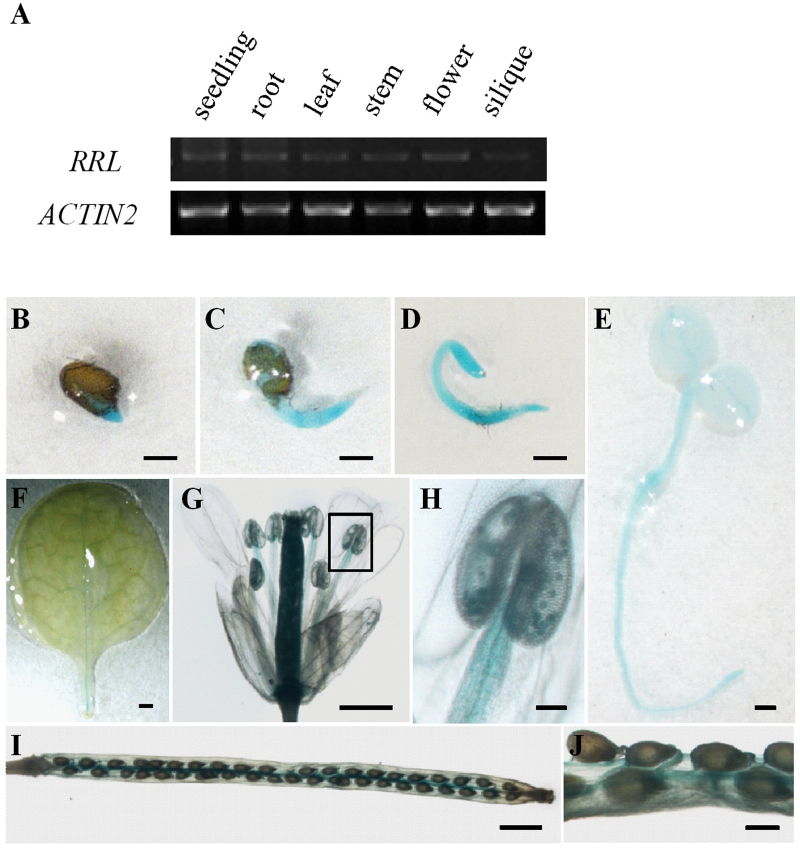
The expression pattern of RRL. (A) Semi-quantitative analysis of *RRL* gene expression in various tissues. *ACTIN2* was used as a loading control. (B–J) *RRL* promoter-driven GUS expression in germinating seeds and developing seedlings (B–D), 4-day-old seedlings (E), rosette leaves (F), flowers (G, H), and siliques (I, J). Scale bars=0.1mm (B, C, D, E, J); 1mm (F, G, I). (H) Magnification of the areas outlined in (G). Scale bar=0.1mm (H). (This figure is available in colour at *JXB* online.)

In order to study the specific function of RRL in seed germination and seedling growth, the expression pattern of the *RRL* gene was next investigated. Semi-quantitative RT-PCR was performed to assess *RRL* gene expression in various tissues including 7-day-old seedlings, roots, rosette leaves, stem, flowers, and siliques. *RRL* was expressed in various tissues (Fig. 4A). In the more detailed GUS staining analyses, 10 independent transgenic *Arabidopsis* lines harbouring the ProRRL–GUS construct were obtained and homozygote T_3_ lines were used for further analyses. Strong GUS staining was detected in germinating seeds and the early developing seedlings (24–96h after seed germination; Fig. 4B–E). In contrast, very weak GUS signal was observed in vascular tissues of rosette leaves (Fig. 4F). Moreover, GUS staining could also be detected in filaments, pollen in flowers (Fig. 4G, H), and false dissepiments and seed stalks in siliques (Fig. 4I, J). The high expression of *RRL* in germinating seeds and developing seedlings suggests a role for RRL in these developmental stages, consistent with the finding that RRL mediates ABA signalling in seed germination and seedling growth.

### The *rrl* mutant exhibits internal vacuolization of mitochondria and reduced ABA-induced ROS production

**Fig. 5.**
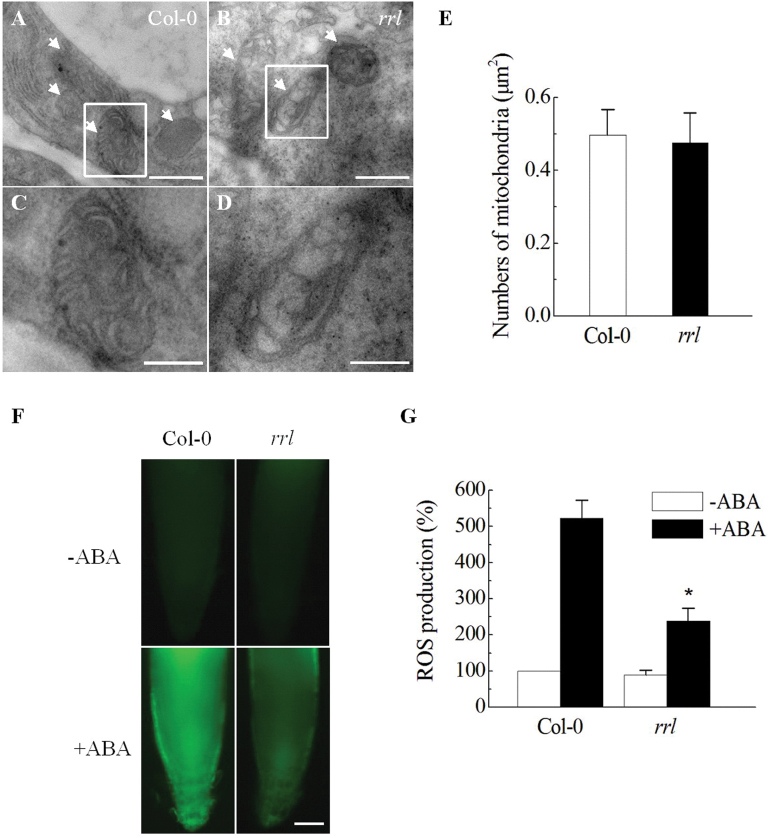
Structure and numbers of mitochondria and ABA-induced ROS production in the *rrl* mutant. (A–D) The mitochondrial structure in Col-0 (A) and the *rrl* mutant (B) was observed by transmission electron microscopy. (C, D) Magnification of the areas outlined in (A, B). Arrows indicate mitochondria. Scale bar=0.5 μm (A, B); 0.25 μm (C, D). (E) Numbers of mitochondria per square micrometre in 5-day-old seedlings of Col-0 and the *rrl* mutant were determined. Values are the means ±SD from three independent measurements. (F) ROS production was detected with the fluorescent dye DCF. Five-day-old seedlings were incubated with 2′,7′-dichlorodihydrofluorescein diacetate (H_2_DCFDA) for 30min. –ABA, ABA-free treatment; +ABA, 50 μM ABA treatment. Scale bar=20 μm. (G) Quantification of ROS levels in Col-0 and the *rrl* mutant before or after 50 μM ABA treatment. (*n*=30; ±SD; *P<0.001 compared with ABA-treated Col-0 plants). The fluorescent intensity in Col-0 with ABA treatment was taken as 100%.(This figure is available in colour at *JXB* online.)

As shown in Fig. 1, RRL is a mitochondria-localized protein in *Arabidopsis*. Next it was examined whether the disruption of the *RRL* gene affects the number and structure of mitochondria. Transmission electron microscopy was used to observe the mitochondria in 5-day-old seedling roots of Col-0 and the *rrl* mutant. A total of 76% of mitochondria in mutant cells exhibited extensive internal vacuolization compared with Col-0 (Fig. 5A–D), but the number of mitochondria per unit of cell area in mutant *rrl* (0.50mm^–2^) was found to be the same as that of Col-0 (0.48mm^–2^) (Fig. 5E). These results suggest that the disruption of the *RRL* gene results in aberrant mitochondrial structure, but does not affect the number of mitochondria (Fig. 5A–E).

Mitochondria produce ROS continuously in metabolically active cells, and the plant hormone ABA could induce ROS production via the mtETC (Miller *et al.*, 2008). Because of the mitochondrial localization of RRL and the aberrant mitochondrial structure in the *rrl* mutant cells, it was next determined whether the mutation of *RRL* led to ABA-induced ROS production. To this end, the ROS levels were compared in 5-day-old seedling roots of Col-0 and the *rrl* mutant with or without ABA treatment. A reduced ABA-induced ROS level was detected after 50 μM ABA treatment for 1h in the *rrl* mutant (Fig. 5F). The ROS level increased ~5.2-fold in Col-0, whereas there was only a 2.7-fold increase in ROS levels in the *rrl* mutant, suggesting that RRL is required for ROS production in response to ABA (Fig. 5G).

### Transcriptional alterations of mtETC-associated gene expression and expression of downstream targets in ABA signalling

**Fig. 6.**
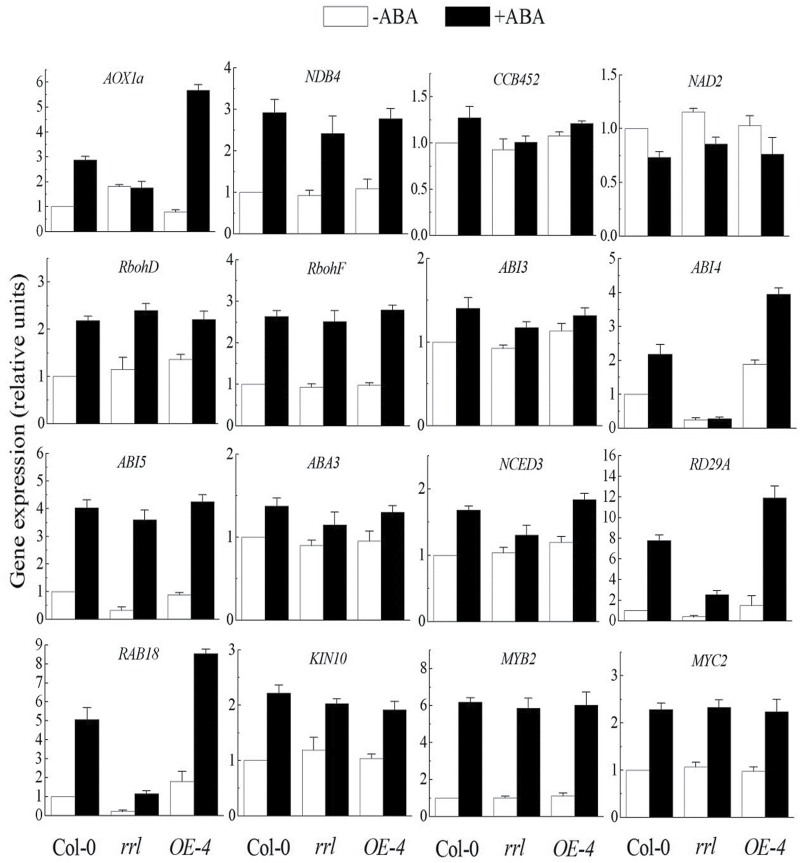
Expression levels of mitochondria-associated genes and ABA-responsive genes in the *RRL* loss-of-function mutant and overexpression line. The expression levels of some mitochondria-associated and ABA-responsive genes were assayed by quantitative RT-PCR with 5-day-old seedlings of Col-0, *rrl*, and *OE-4*. –ABA, ABA-free treatment; +ABA, 50 μM ABA treatment. Gene expression levels were analysed as relative units, with that of ABA-free treated Col-0 plants being taken as 1. Values are the means ±SE of three independent experiments.

The requirement for RRL for maintaining the structure and function of mitochondria (Fig. 5) prompted an assay of the expression of mtETC-associated genes including *AOX1a*, *NDB4*, *CCB452*, and *NAD2* to be carried out. An isoform of alternative oxidase AOX1a functions as a non-phosphorylating bypass of mitochondrial electron transport, and NDB4 is a alternative type II NAD(P)H dehydrogenase without proton translocation activity in the alternative respiratory pathway (Melo *et al.*, 2004). CCB452 is associated with biogenesis of cytochrome *c* (van Dongen *et al.*, 2011) and *NAD2* encodes a subunit of NAD(P)H dehydrogenase in mitochondrial respiratory chain complex I (Heazlewood *et al.*, 2003). Expression levels of genes were quantitatively analysed after the 5-day-old seedlings were subjected to ABA for 6h (Fig. 6). The expression of mtETC-associated genes including *CCB45*, *NDB4*, and *NAD2* was not altered by disruption or overexpression of *RRL* in both the absence and presence of the ABA treatments, except for *AOX1a* (Fig. 6). In this report, ABA-induced expression of *AOX1a* was found in Col-0 plants as described previouly (Giraud *et al.*, 2009). It should be noted that *AOX1a* expression was increased in *the rrl* mutant in the absence of the ABA treatments, but the ABA induction was affected by mutation of *RRL*. Overexpression of *RRL* significantly enhanced the expression level of *AOX1a* in response to ABA (Fig. 6). Because *AOX1a* induction also serves as a marker of mitochondrial retrograde responses in *Arabidopsis* (Rhoads and Subbaiah, 2007), this result suggests that *RRL* is involved in the retrograde signalling between the mitochondria and nucleus in response to ABA.

To understand whether the ABA-insensitive phenotypes of the *rrl* knockout mutant and ABA-hypersensitive phenotypes of the transgenic overexpression lines in seed germination and seedling growth (Figs 2, 3) were due to altered ABA signalling, the expression levels of the following ABA-responsive genes were also tested: *RbohD* and *RbohF* (Kwak *et al.*, 2008), *ABI3* (Giraudat *et al.*, 1992), *ABI4* (Finkelstein *et al.*, 1998), *ABI5* (Finkelstein and Lynch, 2000), *ABA3* (Léon-Kloosterziel *et al.*, 1996), *NCED3* (Iuchi *et al.*, 2001), *RD29A* (Yamaguchi-Shinozaki and Shinozaki, 1994), *RAB18* (Lang and Palva, 1992), *KIN10* (Baena-González et al, 2007), and *MYB2* and *MYC2* (Abe *et al.*, 2003). Results from gene expression assays showed that disruption or overexpression of *RRL* did not affect the expression of *RbohD*, *RbohF*, *ABI3*, *ABI5*, *ABA3*, *NCED3*, *KIN10*, *MYB2*, and *MYC2* in both the absence and presence of ABA treatment. *ABA3* and *NCED3* are two key genes which function in ABA biosynthesis. Similar expression levels of *ABA3* and *NCED3* in Col-0, the *rrl* mutant, and the overexpression line in response to ABA indicated that *RRL* gene expression did not alter the ABA content. Disruption of *RRL* down-regulated expression of *ABI4*, *RD29A*, and *RAB18* in both the absence and presence of ABA treatments; in contrast, overexpression of *RRL* amplified the ABA-induced expression effects on these downstream targets in ABA signalling (Fig. 6). The AP2-type transcription factor ABI4 plays a positive role in ABA signal transduction (Finkelstein *et al.*, 1998). It binds to and keeps the AOX1a promoter constitutively repressed, and ABA can lift the repression to stimulate *AOX1a* gene expression by mediating mitochondrial retrograde signalling (Giraud *et al.*, 2009). The expression of *ABI4* was significantly deceased and ABA-induced *AOX1a* expression was impaired in the *rrl* mutant in the presence of ABA treatments, suggesting that ABI4 is required for RRL-mediated ABA signalling and mitochondrial retrograde regulation. Taken together, these results on gene expression suggested that the altered ABA responsiveness in germination and seedling growth of the *rrl* mutant and the *OE-4* overexpression line is probably due to ABA signalling modulated by ABI4 expression and ABI4-mediated mitochondrial retrograde regulation.

### RRL mediates ABA signal transduction by regulation of ABI4 expression

**Fig. 7.**
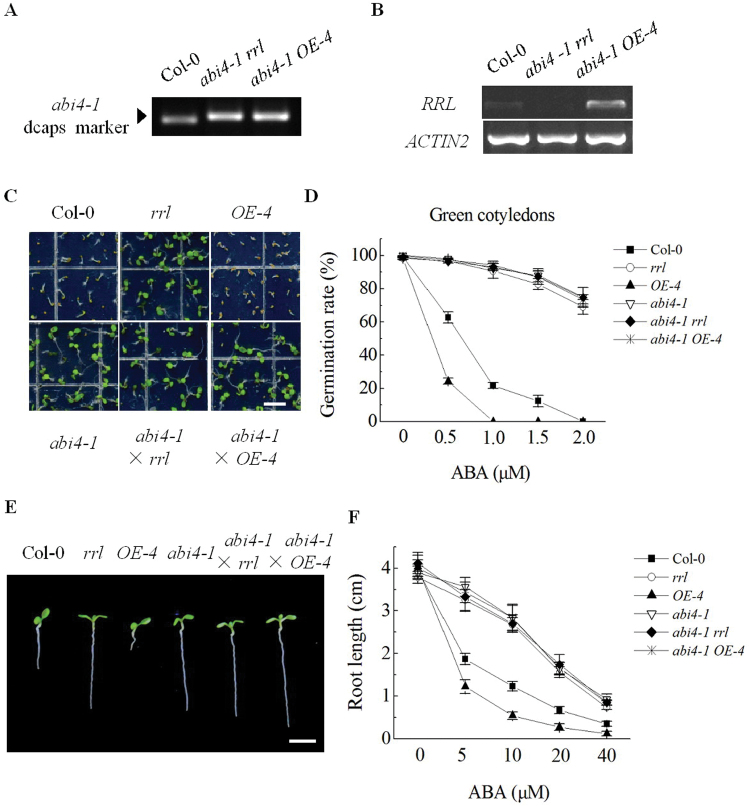
Genetic analysis of the double mutant *abi4-1 rrl* and the hybrid *abi4-1 OE-4*. (A, B) F_2_ generation seedlings of crossed plants were subjected to single nucleotide polymorphism (SNP) identification to isolate *abi4* mutation loci (A) and semi-quantitative RT-PCR to determine expression of *RRL* (B). *ACTIN2* was used as the internal control. (C) Seeds were sown on MS medium supplemented with 1.0 μM ABA. Photographs were taken at day 5 after stratification. Scale bar=0.5cm. (D) Seeds were sown on MS medium supplemented with the indicated concentration of ABA. Germination rates (%) were analysed at day 5 after stratification. Values are the means ±SE of three independent experiments (*n*=100). (E) Seedlings grew for 10 d after transfer from MS medium to MS medium supplemented with 10 μM ABA. Seedlings were transferred from ABA-free medium to ABA-containing medium 48h after stratification. Scale bar=0.5cm. (F) Primary root lengths were assayed after seedlings were transferred to MS medium supplemented with 0, 5, 10, 20, or 40 μM ABA for 10 d. Values are the means ±SE of three independent experiments (*n*=50). (This figure is available in colour at *JXB* online.)

To determine whether *ABI4* gene expression is necessary for the RRL-mediated ABA signalling pathway, mutant *abi4-1* (maternal) was crossed with *rrl* (paternal) or the overexpression line *OE-4* (paternal). The *abi4-1* mutation locus was isolated by a dCAPS marker and *RRL* expression was analysed by semi-quantitative RT-PCR in the F_2_ generation seedlings of crossed plants (Fig. 7A, B). In the seed germination assay, the *abi4-1* mutant showed an ABA-insensitive phenotype, consistent with a previous study (Finkelstein *et al.*, 1998). The double mutant *abi4-1 rrl* and hybrid *abi4-1 OE-4* showed a similar ABA-insensitive phenotype to *abi4* mutants when 1.0 μM ABA was applied (Fig. 7C). Furthermore, germination rates with 0, 0.5, 1.0, 1.5, and 2.0 μM ABA concentrations were assessed by scoring the open green cotyledons at day 5 after stratification. The germination rates of the double mutant *abi4-1 rrl* and hybrid *abi4-1 OE-4* were significantly higher than those of Col-0, which were comparable with those of *rrl* and *abi4-1* mutants. In contrast, the germination rates of the overexpression line *OE-4* were drastically reduced compared with Col-0 (Fig. 7D). The length of the primary root of seedling plants grown on MS medium containing the indicated concentration of ABA for 10 d were also measured. The primary root lengths of the double mutant *abi4-1 rrl* and hybrid *abi4-1 OE-4* were also comparable with those of mutant *rrl* and mutant *abi4-1* when the seedlings grew on MS medium containing the indicated concentrations of ABA, whereas the primary root elongation of the overexpression line *OE-4* was significantly inhibited by the ABA treatments compared with Col-0 (Fig. 7E, F). Taken together, the data suggest that ABI4 plays a role downstream of RRL in ABA signalling during seed germination and primary root growth.

**Fig. 8.**
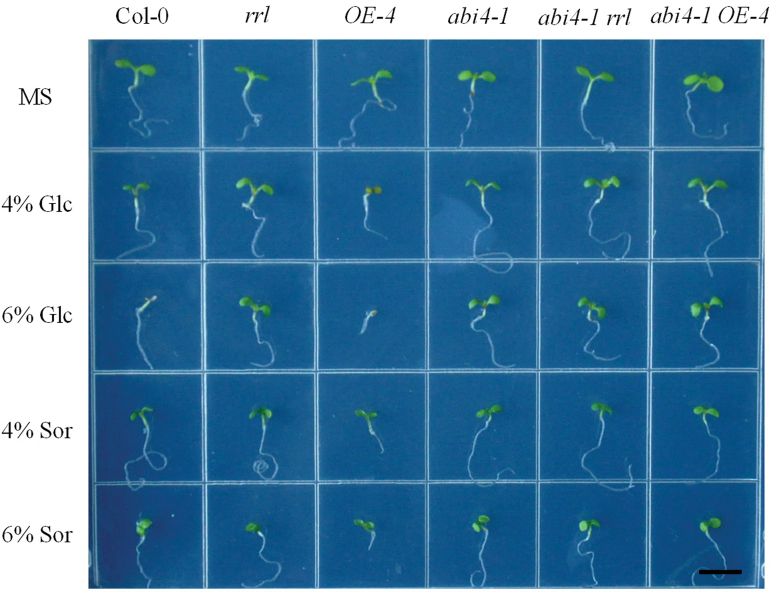
Responses to glucose of the *rrl* knockout mutant, the *OE-4* overexpression line, the *abi4-1 rrl* double mutant, and the *abi4-1 OE-4* hybrid. Glucose hypersensitivity of the RRL overexpression line *OE-4* (4–6% glucose) and glucose insensitivity of the *rrl* knockout mutant, *abi4-1* mutant, *abi4-1 rrl* double mutant, and *abi4-1 OE-4* hybrid (4–6% glucose) in early seedling development. Sor, sorbitol osmotic control; Glc, glucose. Scale bar=0.5cm. (This figure is available in colour at *JXB* online.)

Previous findings have revealed that ABI4 is involved in the ABA-mediated glucose response. Early seedling development is inhibited in the wild type in response to sugars. However, the *abi4* mutant displays expanded green cotyledons and true leaves on 6% glucose (Arenas-Huertero *et al.*, 2000; Huijse, *et al.*, 2000). Thus the responses of the *rrl* mutant, the *OE-4* overexpression line, the *abi4-1* mutant, the *abi4-1 rrl* double mutant, and the *abi4-1 OE-4* hybrid to glucose were next tested. The results showed that the *rrl* mutant, the *abi4-1 rrl* double mutant, and the *abi4-1 OE-4* hybrid exhibited the same reduced sensitivity to sugar as the *abi4-1* mutant on MS medium supplemented with 4% and 6% glucose. In contrast, the *OE-4* overexpression line displayed increased sensitivity to 4% and 6% glucose compared with Col-0 (Fig. 8). Sorbitol at 4% and 6% was used as the osmotic control in this experiment. These results suggest that disruption of *RRL* renders seedlings sugar insensitive and RRL may participate in ABI4-mediated sugar signalling.

## Discussion

Mitochondrial proteome studies have discovered that >1000 proteins are present in plant mitochondria. A great number of mitochondria-localized proteins regulated by anterograde (nuclear to mitochondrial) or retrograde (mitochondrial to nuclear) signalling are involved in growth, development, and stress signalling pathways in plant. Therefore, mitochondria are important hubs of the internal and external signals in the cells (Ng *et al.*, 2014). Plants are able to alter the endogenous levels of hormones such as ABA in response to abiotic and biotic stresses, which can target mitochondria directly and lead to mitochondrial dysfunction. Mitochondria in turn play vital roles in transmission and execution of the signals during overall plant stress responses. However, the molecular mechanisms of mitochondrial responses to a variety of stresses are still unclear, although a few components in the signalling pathway have begun to be found (Finkelstein *et al.*, 2002; Rhoads and Subbaiah, 2007; Ng *et al.*, 2014). In this study, the function of an *Arabidopsis* mitochondria-localized protein RRL in the ABA signalling pathway has been identified. RRL is expressed in germinating seeds and early developing seedlings, and plays a positive role in ABA signal transduction during seed germination and seedling growth. Moreover, RRL-mediated ABA signalling is regulated by mitochondrial retrograde regulation mediated by the AP2 transcription factor ABI4.

### RRL positively regulates ABA signalling during seed germination and seedling growth

This study revealed that RRL was a positive regulatory factor in ABA signalling during seed germination and primary root growth (Figs 2, 3). However, the expression of *RRL* did not alter the response of plants to drought stress (see Supplementary Fig. S3 at *JXB* online). The expression pattern of *RRL* was further determined in various tissues. High expression levels of *RRL* were found in germinating seeds and developing seedlings; however, only very low expression levels of *RRL* were found in vascular tissues of rosette leaves, showing a close correlation to the functions of RRL in ABA signalling in seed germination and seedling growth (Fig. 4). In order to investigate how RRL regulates ABA signalling, the expression levels of some ABA-responsive genes were next examined. The data from quantitative RT-PCR experiments showed that the expression levels of the marker genes of ABA signalling such as *RD29A* (Yamaguchi-Shinozaki and Shinozaki, 1994) and *RAB18* (Lang and Palva, 1992) were significantly reduced in the *rrl* mutant but elevated in the *OE-4* overexpression line with ABA treatment; in contrast, *RbohD*, *RbohF* (Kwak *et al.*, 2008), *KIN10* (Baena-González et al, 2007), and *MYB2* and *MYC2* (Abe *et al.*, 2003) displayed similar expression levels in both the *rrl* mutant and the *OE-4* overexpression line in the presence or absence of ABA treatment (Fig. 6). These results suggest that RRL mediates ABA signalling but not through the regulation of the above components. As shown in Figs 2–4, the high expression levels of *RRL* and altered plant responses to ABA were all found in germinating seeds and developing seedlings; thus the expression of *ABI3* (Giraudat *et al.*, 1992), *ABI4* (Finkelstein *et al.*, 1998), and *ABI5* (Finkelstein and Lynch, 2000), which are three transcription factors playing essential roles in ABA signalling in seeds and seedlings (Finkelstein *et al.*, 2002), was investigated. The results showed that the expression of *ABI4* was significantly down-regulated in the *rrl* mutant and up-regulated in the *OE-4* overexpression line after ABA treatment, whereas the expression of *ABI3* and *ABI5* was not affected in either the *rrl* mutant or the *OE-4* overexpression line (Fig. 6). These results suggest that it is probable that ABI4 acts downstream of RRL-mediated ABA signalling during seed germination and seedling growth. Genetic analysis was next performed to confirm that RRL and ABI4 function in the same pathway and that ABI4 is a downstream component of RRL-mediated ABA signal transduction (Fig. 7).

Interestingly, in addition to the ABA-insensitive phenotype of the *rrl* mutant, its insensitive phenotype to high sugar levels was also found. The *abi4-1* mutant was insensitive to 6% glucose as described previously, and the *rrl* mutant showed the same response as the *abi4-1* mutant (Fig. 7). In addition, the results from genetic analysis suggest that ABI4 also acts downstream of RRL in sugar signalling (Fig. 7). Previous findings have demonstrated that sugar signalling pathway interacts with the ABA signalling pathway, and common signalling components shared in the both signalling pathways have been reported by intensive studies (Cheng *et al.*, 2002; Rolland *et al.*, 2006). In this report, RRL was found to be such a common component functioning in both the sugar signalling pathway and the ABA signalling pathway (Figs 7, 8).

### The function of RRL in mitochondrial retrograde regulation

Anterograde regulation is a top-down signalling pathway from the nucleus to the organelle, whereas retrograde regulation is a bottom-up signalling pathway from the organelle to the nucleus (Leister, 2005; Liu and Butow, 2006). Retrograde regulation takes place when endogenous or external stimuli alter the functioning of organelles and signals originating from the organelles in response control nuclear gene expression. Modulated anterograde regulation can in turn affect the functioning of organelles (Butow and Avadhani, 2004; Pesaresi *et al.*, 2007). Abiotic stress, biotic stress, or mutation results in a dysfunctional mETC in mitochondria, and the expression of AOXs and alternative NAD(P)H dehydrogenases was subsequently induced by mitochondrial retrograde regulation, which can compensate ROS production to regain the redox homeostasis and ROS signalling. *AOX1a* encodes an isoform of AOX and functions as a marker for mitochondrial retrograde regulation. A previous study has revealed that ABI4 keeps *AOX1a* expression repressed by binding of a CCAC *cis*-acting element of the *AOX1a* promoter, and the repression can be lifted by ABA (Giraud *et al.*, 2009).

In the present study, it was found that RRL was localized to mitochondria (Fig. 1), and disruption of *RRL* impaired the normal structure of mitochondria (Fig. 5A–D). The aberrant structure has an impact on the functioning of mitochondria such as the reduced production of ABA-stimulated ROS (Fig. 5F, G). MtROS originated from the mETC which is localized at the inner mitochondrial membrane. Two partially overlapping respiratory pathways are reported for mETC, namely the cytochrome respiratory pathway with cytochrome oxidase (COX) as the terminal oxidase and the alternative respiratory pathway with alternative oxidase (AOX) as the terminal oxidase (Finnegan *et al.*, 2004; McDonald and Vanlerberghe, 2004). Interestingly, a putative C-terminal transmembrane domain was predicted in the mitochondria-localized RRL protein sequence (http://smart.embl-heidelberg.de/smart/show_motifs.pl), implying its possible relationship to the respiratory pathway in mitochondria (see Supplementary Fig. S1B at *JXB* online). Thus the expression of *AOX1a* and *NDB4* associated with the alternative respiratory pathway and *CCB452* and *NAD2* associated with the cytochrome respiratory pathway was examined in the *rrl* mutant and *OE-4* overexpression line with or without ABA treatment. In contrast to the similar expression levels of *NDB4*, *CCB452*, and *NAD2* compared with the wild type (Col-0), an enhanced expression level of *AOX1a* was found in the *rrl* mutant in the absence of ABA treatment. Because a previous report has revealed that *AOX1a* expression is repressed by *ABI4* under normal conditions (Giraud *et al.*, 2009), the elevated *AOX1a* expression might be caused by down-regulated expression of ABI4 in the *rrl* mutant (Fig. 6). It is should be noted, however, that ABA-induced *AOX1a* expression was affected by the disruption of *RRL* after ABA treatment, which would explain the reduced ABA-induced ROS production in the *rrl* mutant (Giraud *et al.*, 2009; Figs 5F, G, 6). Taken together, the present study provides evidence that RRL-mediated mitochondrial retrograde regulation is modulated by the expression of *ABI4* and *AOX1a*. RRL probably plays a positive role in ABA-induced *AOX1a* accumulation, which consequently leads to increased levels of mtROS; the mtROS, as signalling molecules, are capable of mediating ABA signal transduction in seed germination and seedling growth (El-Maarouf-Bouteau and Bailly, 2008; Gomes and Garcia, 2013). The molecular mechanisms of mitochondrial retrograde regulation have not been elucidated to date, and how ROS-stimulated signalling is transmitted to the nucleus to trigger gene expression in mitochondrial retrograde signalling remains unclear. A recent study has revealed that a group of endoplasmic reticulum (ER)-bound NAC transcription factors such as ANAC017 would help to explain how ROS signals can be transmitted in mitochondrial retrograde signalling (Ng *et al.*, 2013). The ER association with actin filaments facilitates a close interaction between the ER and mitochondria, which would set the stage for ROS diffusion over the ER to activate the release of the membrane-bound NAC transcription factors from the ER. The released NAC transcription factors finally migrate into the nucleus to relay the signals, providing the possibility of transmission of ROS signalling. Additionally, a group of WRKY transcription factors such as WRKY40 and WRKY63 also seem to be the responsive regulators in ROS-mediated mitochondrial retrograde signal transduction (Ng *et al.*, 2014). More studies are required to identify the functional components to better understand the mitochondrial retrograde signalling pathway.

In conclusion, the findings in this study demonstrated the positive function of RRL in ABA signalling in *Arabidopsis* seed germination and seedling growth. RRL mediated ABA signal transduction through the downstream regulatory transcription factor ABI4 during seed germination and seedling growth. Moreover, mitochondria-localized RRL probably increased ROS levels in response to ABA, and ROS signals were sequentially transmitted to the nucleus to regulate gene expression in mitochondrial retrograde signalling.

## Supplementary data

Supplementary data are available at *JXB* online.

Figure S1. Analysis of the RRL protein sequence.

Figure S2. Disruption of *RRL* does not cause retarded root growth.

Figure S3. Mutation of *RRL* does not alter the plant response to drought stress.

Figure S4. *rrl* mutants show normal development of guard cells.

Table S1. Primer sequences used for plasmid constructions in this study.

Table S2. Primer sequences used for semi-quantitative and quantitative RT-PCR experiments in this study.

Supplementary Data

## Abbreviations:

ABA: abscisic acid
ABF/AREB: ABA-responsive element binding factor
ABRE: ABA-responsive element
AOX: alternative oxidase
bZIP: basic leucine zipper
COX: cytochrome oxidase
dCAPS: derived cleaved amplified polymorphic sequences
DCF: dichlorofluorescein
GUS: β-glucuronidase
H_2_DCFDA: 2′,7′-dichlorodihydrofluorescein diacetate
mETC: mitochondrial electron transport chain
mtROS: mitochondrial ROS
ROS: reactive oxygen species.
