# Supplementary material for: An Arabidopsis mitochondria-localized RRL protein mediates abscisic acid signal transduction through mitochondrial retrograde regulation involving ABI4
Source: J Exp Bot. 2015 Jul 10;66(20):6431–45. doi: 10.1093/jxb/erv356 (PMC4588890; doi:10.1093/jxb/erv356)
Supplement: Supplementary Data [file supp_66_20_6431__index.html]

An Arabidopsis mitochondria-localized RRL protein mediates abscisic acid signal transduction through mitochondrial retrograde regulation involving ABI4 — An Arabidopsis mitochondria-localized RRL protein mediates abscisic acid signal transduction through mitochondrial retrograde regulation involving ABI4 — Supplementary Data 

# An *Arabidopsis* mitochondria-localized RRL protein mediates abscisic acid signal transduction through mitochondrial retrograde regulation involving ABI4

## Supplementary Data

Data files

- Supplementary Data - Supplementary Data
