# Supplementary material for: An Arabidopsis mitochondria-localized RRL protein mediates abscisic acid signal transduction through mitochondrial retrograde regulation involving ABI4
Source: J Exp Bot. 2015 Jul 10;66(20):6431–45. doi: 10.1093/jxb/erv356 (PMC4588890; doi:10.1093/jxb/erv356)
Supplement: Supplementary Data [file supp_erv356_jexbot150045_file001.pdf]

An *Arabidopsis* mitochondria-localized RRL protein mediates abscisic acid signal transduction though ABI4-invovled mitochondrial retrograde regulation. *Xuan Yao, Juanjuan Li, Jianping Liu, and Kede Liu*

SUPPLEMENTARY DATA

A

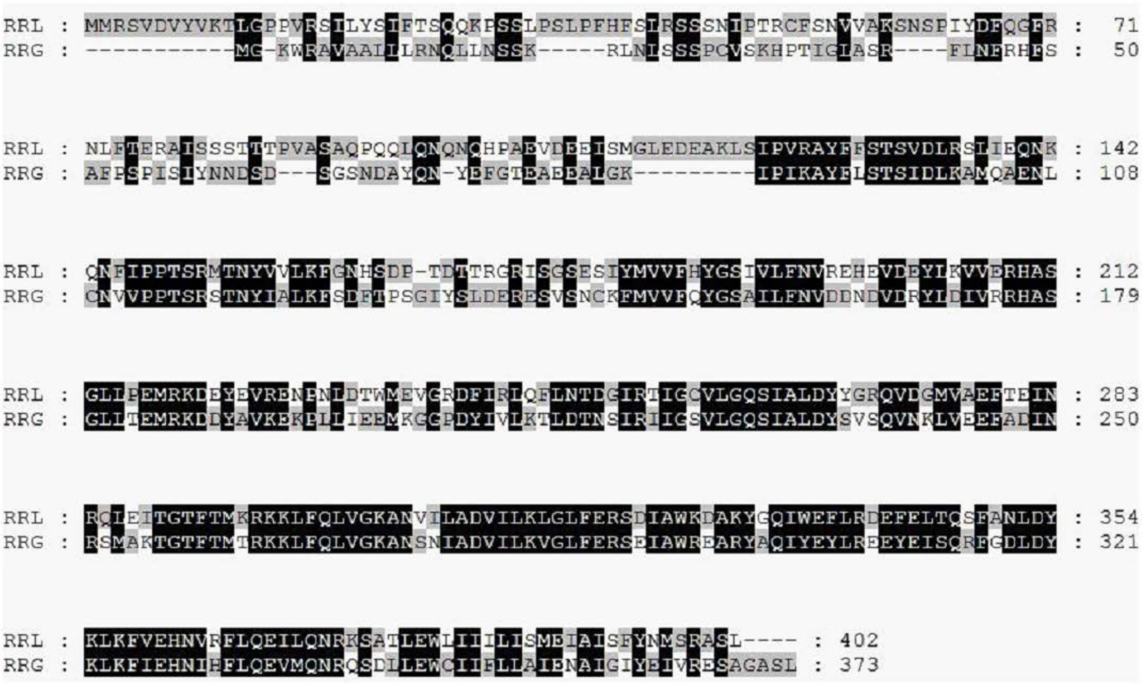

B

1 MMRSVDVYVKTLGPPVRSILYSIFTSQQKPSSLPSLPFHFSLRSSSNIPT

51 RCFSNVVAKSNSPIYDFQGFRNLFTERAISSSTTTPVASAQPQQLQNQNQ

101 HPAEVDEEISMGLEDEAKLSIPVRAYFFSTSVDLRSLIEQNKQNFIPPTS

151 RMTNYVVLKFGNHSDPTDTTRGRISGSESIYMVVFHYGSIVLFNVREHEV

201 DEYLKVVERHASGLLPEMRKDEYEVRENPNLDTWMEVGRDFIRLQFLNTD

251 GIRTIGCVLGQSIALDYYGRQVDGMVAEFTEINRQLEITGTFTMKRKKLF

301 QLVGKANVILADVILKLGLFERSDIAWKDAKYGQIWEFLRDEFELTQSFA

351 NLDYKLKFVEHNVRFLQEILQNRKSATLEWLIIILISMEIAISFYNMSRA

401 SL

Fig. S1. Analysis of RRL protein sequence

(A) The alignments between predicted RRL and RRG protein sequences by using Clustalx and GeneDoc3.2 tools. (B) Deduced amino acid sequence of the RRL protein and prediction of the protein structure. Conserved putative DUF155 domain is underlined, and predicted transmembrane domain is boxed.

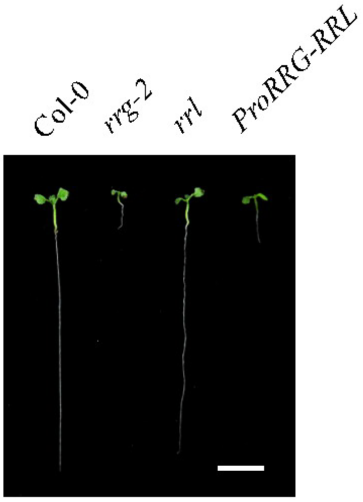

Fig. S2. Disruption of *RRL* does not cause retarded root growth.
Retarded root growth of *rrg-2* mutant (T-DNA insertion mutant of *RRG*, CS390410) and normal root growth of *rrl* mutant. The introduction of ProRRG-RRL in the *rrg-2* mutant background did not recover the retarded root growth phenotype.

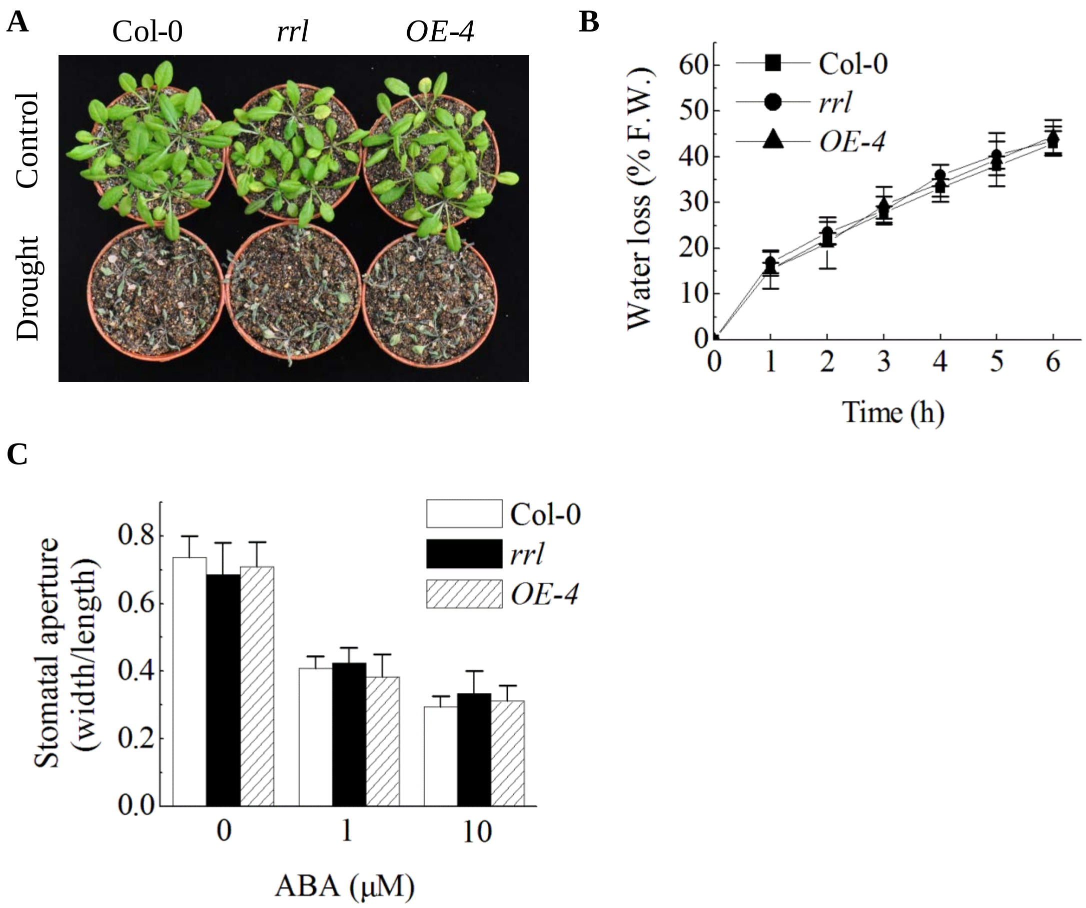

Fig. S3. *RRL* mutation does not alter plant response to drought stress.

(A) 2-week-old Col-0, *rrl* and *OE-4* plants were well watered (Control) or withhold water for 14 days and then rewatered (Drought). The photos were taken on day 1 after rewatering. (B) Transpirational water loss from detached leaves of 4-week-old Col-0, *rrl* and *OE-4* plants at the indicated time points. Water loss rates are indicated as the percentage of the initial fresh weight (% FW). Results are shown as the means ±SD from three replicated samples with five leaves. (C) Stomatal closure assay. Values are means ±SE from three independent experiments (n = 50). The epidermal peels of leaves from 4-week-old Col-0, *rrl* and *OE-4* plants were first incubated in the light for 3 h to induce stomatal opening and then treated with 0, 1.0 and 10 µM ABA for 3 h.

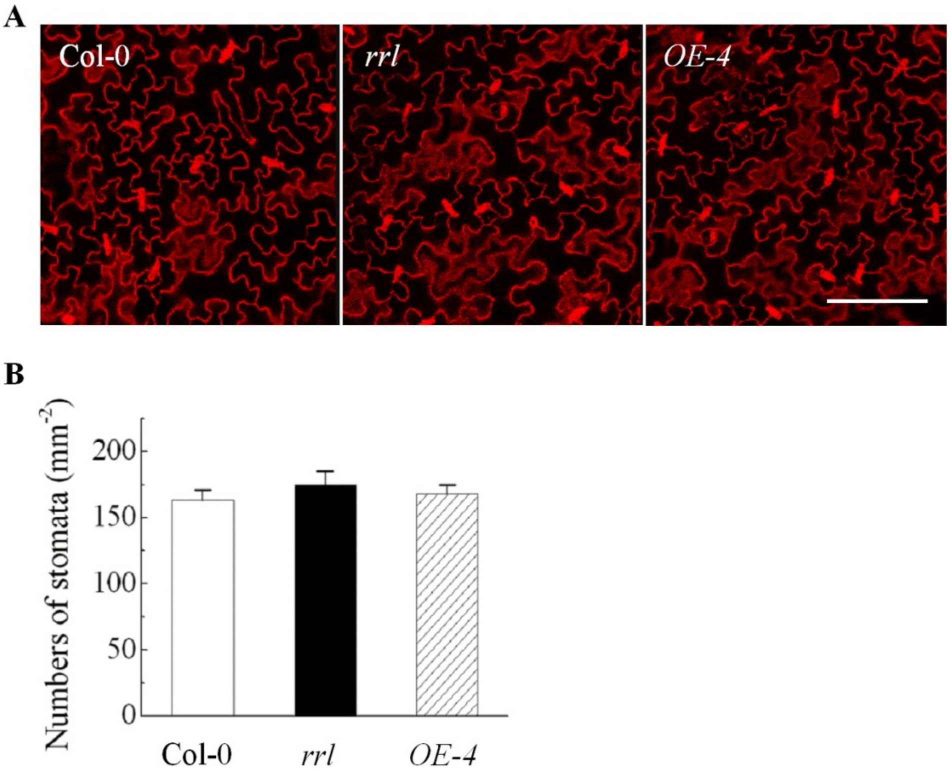

Fig. S4. *rrl* mutants show normal development of guard cells.

(A) The epidermis of the abaxial surface of rosette leaves from Col-0, *rrl* and *OE-4* plants (Bar = 100 μm). (B) Numbers of stomata per square millimeter in the epidermis of rosette leaves of Col-0, *rrl* and *OE-4* plants. Data are the means ±SE from leaves of three individual plants of Col-0, *rrl* and *OE-4*. Three independent counts were carried out on each leaf.

Table S1 Primer sequences were used for plasmids constructions in this study.

ProRRL-RRL	F: 5’-CGCGGATCCGGCCCTGGAGAAATCATGC-3’
	R: 5’-AAAACTGCAGCCTCATTTTGCTTCGTAATTTC-3’
RRL-OE	F: 5’-CCGCCATGGGGCGGCTCTGTTGTTGAT-3’
	R: 5’-CCGCACGTGTCACAAGGATGCCCGAGAC-3’
ProRRL-GUS	F: 5’-CGGGGTACCCTGGGAAGATTCCATCAAGGCCCTG-3’
	R: 5’-AAAACTGCAGTTTGCTTTTTTACACAGAGAGTAAG-3’
RRL-GFP	F:5’-ACGCGTCGACATGATGCGTTCCGTTGACGT-3’
	R: 5’-AAAACTGCAGCAAGGATGCCCGAGACATATTA-3’

Table S2 Primer Sequences were used for semi-quantitative and quantitative RT-PCR experiments in this study.

ACTIN2	F: 5'-ATGGCAGACGGTGAGGATATTCA-3'
	R: 5'-GCCTTTGCAATCCACATCTGTTG-3'
ABI4dcaps	F: 5'- ATGGACCCTTTAGCTTCCCAACATC-3'
	R: 5'- AGTTACCGGAACATCAGTGAGCTCG-3'
RRL	F: 5'-CCGCCATGGGGCGGCTCTGTTGTTGAT-3'
	R: 5'-CCGCACGTGTCACAAGGATGCCCGAGAC-3'
β-ACTIN8	F: 5'-AGTGGTCGTACAACCGGTATTGT-3'
	R: 5'-GAGGATAGCATGTGGAAGTGAGAA-3'
AOX1a	F: 5'-TGGTTGTTCGTGCTGACG-3'
	R: 5'-CACGACCTTGGTAGTGAATATCAG-3'
NDB4	F: 5'-TCCAAGATTCTTCTTCTCAGCAC-3'
	R: 5'-GGATTCGAGTCTGAATACACCA-3'
CCB452	F: 5'-CACATGGAGGAGTGTGCATC-3'
	R: 5'-GTGGGTCCATGTAAATGATCG-3'
NAD2	F:5'-GGATCCTCCCACACATGTTC-3'
	R:5'-GCGAGCAGAAGCAAGGTTAT-3'
RbohD	F: 5'- AACAACAGGTGGCTGTTTACC-3'
	R: 5'-TGTGATTGAGAAAGGATGCC-3'
RbohF	F: 5'-TTCAGTATCCGTGGGCAATA-3'
	R: 5'-CACTCCTGCGAAAGATCAAA-3'
ABI3	F:5'-GGAGCTAGAGGCAAGAGACG-3'
	R:5'-TCAGTTACCTGTAGCGCATGT-3'
ABI4	F:5'-GCTTCCCAACATCAACACAACC-3'
	R:5'-TTGAGCGGAGGAAGTTGATGAG-3'
ABI5	F: 5'-GAGAATGCGCAGCTAAAACA-3'
	R: 5'-GTGGACAACTCGGGTTCCTC-3'
NCED3	F: 5'-TTGATGCTCCAGATTGCTTC-3'
	R: 5'-GGACCCTATCACGACGACTT-3'
ABA3	F: 5'-AAGTCCATGGATCCACACAA-3'
	R: 5'-TTATCATCTGGCACCGGTTA-3'

RD29A	F: 5’-AGGAACCACCACTCAACACA-3’
	R: 5’- GCTCATGCTCATTGCTTTGT-3’
RAB18	F: 5’-AGCTCTAGCTCGGAGGATGA-3’
	R: 5’-CATGATGACCTGGCAACTTC-3’
KIN10	F: 5’-AATTTGATCGCACAGTCTCG-3’
	R: 5’-TGCTGTTAGGAACCCATCTG-3’
MYB2	F:5’-ATGGACCGAGGAAGAAGATG-3’
	R:5’-TTGATGATACCAGAGGAACGA-3’
MYC2	F:5’-GGTGAGAACGACCCGTCTAT-3’
	R:5’-CGTTACCCGGTTCGTTAGAT-3’
